# Investigating the Relationship Between Long Non-Coding RNAs and miR-200 Family Expression in Clear Cell Renal Cell Carcinoma

**DOI:** 10.3390/cancers17193123

**Published:** 2025-09-25

**Authors:** Tanja Čugura, Nina Hauptman, Jera Jeruc, Emanuela Boštjančič

**Affiliations:** Institute of Pathology, Faculty of Medicine, University of Ljubljana, Korytkova 2, 1000 Ljubljana, Slovenia; tanja.cugura8@gmail.com (T.Č.); nina.hauptman@mf.uni-lj.si (N.H.); jera.jeruc@mf.uni-lj.si (J.J.)

**Keywords:** renal cell carcinoma, epithelial–mesenchymal transition, long non-coding RNA, *miR-200* family

## Abstract

**Objectives:** MicroRNAs of the *miR-200* family are recognized as key inhibitors of epithelial-to-mesenchymal transition (EMT). However, there is limited data on the potential regulation of *miR-200* family expression by long non-coding RNAs (lncRNAs) in RCC. **Methods:** We conducted a comprehensive literature and database search to identify lncRNAs that had been already functionally validated as regulators of any member of the *miR-200* family. We analyzed the expression levels of the *miR-200* family and the identified lncRNAs by qPCR. The study included 42 samples of carcinoma and non-carcinoma tissue from 25 RCC patients. In addition, we used RNA sequencing data from The Cancer Genome Atlas (TCGA), encompassing 511 kidney RCC (KIRC) samples, to further analyze the expression of miRNAs and lncRNAs. **Results:** We identified 127 lncRNAs with confirmed regulatory functions, 31 of which were validated in our samples. The majority of lncRNAs, along with all members of the *miR-200* family, showed consistent downregulation in carcinoma tissues compared to non-carcinoma tissues. We observed a significant correlation between the expression of at least one member of the *miR-200* family and 17 lncRNAs. In particular, three lncRNAs (*MALAT1*, *OIP5-AS1*, and *LINC00467)* showed a correlation with the expression of all members of the *miR-200* family. Our results were at least partially confirmed in KIRC samples from the TCGA dataset. **Conclusions:** Our results suggest that the expression of the *miR-200* family in RCC might be at least partially influenced by lncRNAs. Based on our cohort of samples, *MALAT1*, *OIP5-AS1*, and *LINC00467* appear to be potentially important contributors to RCC development.

## Simple Summary

Non-coding RNAs (ncRNAs) are a class of RNA molecules that do not encode proteins and primarily serve regulatory functions. They are categorized by length into small ncRNAs (e.g., microRNAs) and long non-coding RNAs (lncRNAs). The microRNAs of the *miR-200* family are well-known inhibitors of epithelial-to-mesenchymal transition (EMT). In our previous studies, we observed *miR-200* family downregulation in renal cell carcinoma (RCC). However, there is limited data regarding the regulation of *miR-200* family expression by lncRNAs in RCC. Our results indicate that *miR-200* family expression in RCC correlates at least in part with the expression of lncRNAs. This work emphasizes the significance of the complex network of different classes of ncRNAs that may contribute to the development of RCC and could be crucial for establishing new biomarkers and for developing new treatment modalities for advanced, incurable forms of RCC.

## 1. Introduction

Non-coding RNAs (ncRNAs) are a class of RNA molecules that do not encode proteins. Less than 5% of the human genome is actively transcribed into messenger RNAs (mRNAs) and protein-coding genes, while the remainder consists of non-coding genes, known as ncRNAs [1]. Although primarily regulatory in function, many ncRNAs have been identified as potential tumor suppressors and/or oncogenes, exhibiting greater specificity for cell and tissue types compared to mRNAs [2,3,4,5]. ncRNA-mediated gene silencing plays a crucial role in regulating biological processes essential for cellular homeostasis. The discovery of ncRNAs significantly enhanced our understanding of various physiological and pathological processes. Functional analyses of many ncRNAs have revealed their key roles in cell cycle regulation, epigenetic regulation, genomic imprinting, cell differentiation, and apoptosis, as well as in various pathologic processes including carcinogenesis. They influence the invasiveness and progression of different carcinomas by regulating a variety of processes, including epithelial–mesenchymal transition (EMT) [6,7,8,9].

ncRNAs can be classified into two main categories: small RNAs, which are less than 200 nucleotides (nt), and long non-coding RNAs (lncRNAs), which are more than 200 nt and extend up to 100 kb [10]. Among small RNAs, microRNAs (miRNAs), typically ranging from 20 to 26 nt, are the most extensively studied, functioning mainly as post-transcriptional regulators of gene expression. Unlike mRNAs, miRNAs lack a 5′ cap and a poly-A tail [11]. They can be expressed as single genes, in gene clusters, or from introns of both protein-coding and non-protein-coding genes, such as lncRNAs [12].

lncRNAs can exhibit similarities to both miRNAs and mRNAs. While lncRNAs share several characteristics with protein-coding RNAs (mRNAs), they lack the open reading frame, exhibit lower sequence conservation, and are expressed at lower levels [13,14]. Based on their loci of origin, lncRNAs can be classified as intergenic/intronic, sense/antisense, bidirectional, or overlapping with protein-coding genes or other ncRNAs [15]. Mechanistically, various lncRNAs function as co-transcriptional regulators of protein-coding genes and play roles in diverse biological and developmental pathways [16]. Studies have shown that approximately 20–30% of lncRNAs can guide specific epigenetic enzymes to their specific region within the genome by physically interacting with lncRNAs and these regions [17]. By binding to chromatin-modifying proteins, lncRNAs can modulate chromatin states, thereby influencing gene expression related to DNA repair and cell adhesion [4,18]. Additionally, by interacting with promoters and transcription factors, they can directly affect the transcription of nearby genes. In cancer cells, these processes can become dysregulated, highlighting the potential role of some lncRNAs in modulating cellular transformation [16].

EMT is considered one of the primary mechanisms of cellular transformation that drives aggressive behavior in advanced cancers. During EMT, epithelial cells acquire mesenchymal traits, reorganize their cytoskeleton, and alter their polarity, transforming into motile, spindle-shaped cells. Key molecular features of EMT include the elevated expression of canonical EMT-transcriptional factors, a cadherin switch from E-cadherin to N-cadherin, and downregulation of the *miR-200* family. The *miR-200* family has been identified as a potent suppressor of EMT through the translational repression of target mRNAs. Our previous research [19] has indicated that EMT and the downregulation of *miR-200* are involved in the development and progression of renal cell carcinoma (RCC), one of the most common urological malignancies with a rising incidence rate [20]. Development of metastases is observed in up to 30% of patients with localized disease, posing significant challenges regarding treatment. Moreover, certain RCC subtypes, such as sarcomatoid renal cell carcinoma (sRCC), are particularly aggressive [21]. Biallelic inactivation of the tumor suppressor gene *VHL* is the predominant genetic alteration in most sporadic clear cell RCCs (ccRCCs). Consequently, inactivation of VHL leads to the stimulation of EMT activation due to accumulation hypoxia-inducible factors (HIFs) [22,23]. However, the exact relationships between *miR-200* signaling pathways and the molecular pathways governing EMT remain complex and not fully understood.

Additionally, various lncRNAs can target specific miRNAs, thereby suppressing or activating their interaction with target mRNAs. This interaction might promote or inhibit EMT and tumor progression. Some lncRNAs targeting the *miR-200* family have previously been studied in ccRCC, including *MALAT1*, *GIHCG*, etc. (reviewed in [24]). The present study aimed to systematically summarize all lncRNAs that have been functionally validated trough in vitro and in vivo experiments as regulators of *miR-200* family expression in various pathological and physiological processes. Additionally, the expression of the identified lncRNAs was analyzed in early and advanced ccRCC, as well as in sRCC, and their levels correlated with the expression of the *miR-200* family. The aim was to identify lncRNAs that might also be potential regulators or targets of the *miR-200* family in RCC.

## 2. Materials and Methods

### 2.1. Patients and Tissue Samples

In our retrospective study, we examined 25 patients diagnosed with renal cell carcinoma, all of whom underwent surgical treatment between 2016 and 2022. The exclusion criteria were as follows: none of the patients had received neoadjuvant therapy, including chemotherapy, radiotherapy, or renal artery embolization prior to surgery. Tumor staging was conducted based on the latest AJCC/UICC TNM staging system for renal tumors [25].

Tumors were graded by two pathologists (JJ and TČ) according to the 2016 edition of the ISUP/WHO pathological grading criteria for renal cell carcinoma [26]. In cases of sRCC, the sarcomatoid component constituted between 30% and 90% of the tumor mass, while the carcinomatous component was ccRCC in all cases of sRCC.

Post-nephrectomy, resection specimens were processed following standard histopathological protocols: fixation in 10% neutral buffered formalin for 24 h, after which representative tissue samples were obtained from both the tumor mass and macroscopically unremarkable renal cortex. These samples were subsequently embedded in paraffin (formalin-fixed paraffin-embedded, FFPE), sectioned into 3–4 µm thick slices, and stained with hematoxylin and eosin for microscopic evaluation.

All slides were subsequently re-examined by a urologic pathology specialist. Patients were divided into three groups: pT1 ISUP/WHO grade II ccRCC (i.e., early ccRCC); pT3 ccRCC with renal vein invasion (i.e., advanced ccRCC); and sRCC.

In the early ccRCC group, representative slides were selected of both the tumor and adjacent non-neoplastic renal cortical tissue. For the sRCC group, slides were chosen that represented the sarcomatous (sRCC-Sa) and the carcinomatous component (sRCC-Ca), as well as the non-neoplastic renal cortical tissue. Tumor cells in all ccRCC and sRCC-Ca cases displayed a characteristic epithelioid morphology, while sRCC-Sa tumor cells showed a distinct spindle-shaped morphology. In advanced ccRCC, representative slides of the tumor center (TC), tumor periphery (TP), renal vein tumor thrombus (VTT), and adjacent non-neoplastic renal cortical tissue were selected. Each type of tumor tissue (early RCC; TC, TP, VTT of advanced ccRCC, sRCC-Ca, and sRCC-Sa) and adjacent non-neoplastic renal cortical tissue was represented by 6 biological replicates (Table S1).

Paraffin blocks corresponding to all slides were retrieved from the archives of the Institute of Pathology, Faculty of Medicine, University of Ljubljana. For molecular analysis, three to five tissue cores (0.6 mm un diameter) were obtained from paraffin blocks: from the TC of both ccRCC (with and without renal vein invasion), the TC of both components of sRCC, the non-neoplastic kidney, TP and VTT of ccRCC with renal vein invasion.

### 2.2. lncRNA Search

To identify lncRNAs that were potential regulators of the *miR-200* family, we performed a search of publications in PubMed using the following key words: “lncRNA & *miR-200*”, “lncRNA & *miR-200a*”, “lncRNA & *miR-200b*”, “lncRNA & *miR-200c*”, “lncRNA & *miR-141*”, and “lncRNA & *miR-429*”. The resulting abstracts were screened for the functionally validated interaction and regulation of the *miR-200* family by lncRNAs irrespective of the cell line or disease context. Studies that, based on abstract screening, did not report a functionally validated lncRNA–*miR-200* family pair or axis were excluded.

### 2.3. Database Search for miRNA-lncRNA Interactions

To systematically catalog interactions between the *miR-200* family (*miR-200a, miR-200b*, *miR-200c*, *miR-141*, and *miR-429*) and lncRNA, we queried three complementary resources that curate RNA–RNA interactions: RNA In-teractome [27], DIANA-LncBase v3 [28], and ENCORI (miRNA–ncRNA module) [29]. Searches were performed for organisms restricted to Homo sapiens and RNA interaction where available. Databases and access dates were as follows: RNA Interactome (http://www.rnainter.org/, accessed on 26 August 2025), DIANA-LncBase v3 (https://diana.e-ce.uth.gr/lncbasev3, accessed on 26 August 2025), and ENCORI miRNA-lncRNA module (https://rnasysu.com/encori/agoClipRNA.php?source=lncRNA, accessed on 26 August 2025). For each miRNA, we retrieved all reported lncRNA targets.

In DIANA-LncBase v3, we retained all reported pairs; in RNA Interactome, we excluded pairs with a score < 0.0001; and in ENCORI, we kept only pairs where the predicted target site overlapped an Ago protein binding site.

We ran three intersection analyses: (i) per-miRNA intersections (lncRNAs present in all three databases for a given miRNA), (ii) within-database pan-miR-200 sets (lncRNAs reported for all five *miR-200* family members within the same database), and (iii) a global intersection spanning all databases and all *miR-200* family members.

### 2.4. RNA Isolation from FFPE Tissue Samples

Three to five 0.6 mm punches from FFPE blocks of each representative region were used for the isolation procedure. Additional slides were cut from punched FFPE blocks, HE-stained, and evaluated by pathologist to ensure that accurate regions were punched for isolation. All reagents were from Thermo Fisher Scientific (Austin, TX, USA), except ethanol (Merck KGaA, Darmstadt, Germany) and the xylene (SigmaAldrich; Merck KGaA, Darmstadt, Germany), which was used as a deparaffinization solution. Manual RNA isolation was performed using the MagMAX FFPE DNA/RNA Ultra kit (Thermo Fisher Scientific, Austin, TX, USA) following the manufacturer’s protocol with one modification, which was overnight digestion in protease. The concentration and purity of the isolated RNA were assessed using a NanoDrop-One (ThermoFisher Scientific; Foster City, CA, USA), measuring wavelengths at 230, 260, and 280 nm.

For quality control, amplification of *GAPDH* (Hs_GAPDH_vb.1_SG, 100 bp, Qiagen) using SYBR Green technology was performed. First, RNA from samples was reverse-transcribed (RT) as described below, and 1 µL of resulting cDNA was used in 10 µL quantitative real-time PCR (qPCR). All samples in this study underwent this initial quality control, and those that would not amplify (Cq < 35) were excluded from further testing.

### 2.5. Analysis of Expression of miR-200 Family

#### 2.5.1. Reverse Transcription (RT) of miRNAs

The miRCURY LNA RT Kit (Qiagen; Hilden, Germany) was used for RT of miRNAs according to the manufacturer’s protocol. UniSp6 was added as spike-in RNA to measure the success of the RT procedure by its subsequent quantification. RT was performed in 10 µL reaction master mix with 10 ng of RNA in 6.5 µL, 0.5 µL UniSp6 Spike-in 1 µL miRCURY RT Enzyme Mix 10× and 2 µL of miRCURY RT Reaction Buffer 5×. The reaction conditions were as follows: 42 °C for 60 min, then heat inactivation of reverse transcriptase at 95 °C for 5 min, followed by immediate cooling to 4 °C.

#### 2.5.2. Quantitative Real-Time PCR (qPCR)

A pre-designed mixture of specific primers for measuring the expression of the *miR-200* family (*miR-141-3p*, *miR-200a-3p*, *miR-200b-3p*, *miR-200c-3p*, and *miR-429)* was used in qPCR with SYBR Green. As reference genes (RGs), *miR-28*, *miR-103a-3p*, and *miR-106a-5p* were used, as suggested by other studies [30]. The 10 µL reaction included 3 µL of diluted (1:60) cDNA, 5 µL of miRCURY LNA SYBR GREEN Kit, 1 µL of miRCURY LNA miRNA PCR Primer Assay 10×, and 1 µL of PCR-grade H_2_O. Rotor Gene Q was used to perform amplification of miRNAs with the following thermal cycling conditions: 95 °C for 2 min, 40 cycles at 95 °C for 10 s, and 56 °C for 1 min; melting curve analysis was performed after amplification to confirm the specificity of amplicons. All qPCR reactions were performed in duplicates. The efficiency of the *miR-200* family and RG amplification was obtained from a previous study [19].

### 2.6. Analysis of Expression of lncRNAs

#### 2.6.1. Reverse Transcription (RT) for lncRNAs

Samples were reversely transcribed using M-MLV Reverse transcriptase (Thermo Fisher Scientific, Waltham, MA, USA) as reported previously [31]. RT was performed by mixing 6 µg of random hexamers and a maximal quantity of RNA and incubated at 65 °C for 10 min. The reaction was followed by adding the RT mix, which included 250 U of M-MLV RT, 10 mM DTT, First Strand Buffer 1×, 1 mM dNTP, 4.5 mM MgCl_2_ and 4 U RNase inhibitor. The volume of RT reaction was 20 µL, and the reaction was run at 25 °C for 10 min, 37 °C for 60 min, and 70 °C for 15 min.

#### 2.6.2. Quantitative Real-Time PCR (qPCR) and Probes

According to initial quality control with *GAPDH*, we chose TaqMan probes (Thermo Fisher scientific, Waltham, MA, USA) for analysis of the expression of lncRNAs and RGs by qPCR to obtain amplicons with a length below 100 bp. mRNAs that were found to be suitable for expression analyses in RCC in previous studies including ours [32] were used as reference genes (*ACTB*, *HPRT1*, *RPL13A*, *SDHA*, and *B2M*) in expression analysis of lncRNAs. All mRNA qPCR analyses were performed in duplicates using a Quant Studio 7 Pro (Thermo Fisher Scientific; Foster City, CA, USA), with signal collection at the endpoint of each cycle.

In the qPCR reaction, 4.5 µL of diluted cDNA (1.4–42 ng) was used in a 10 µL total reaction volume containing 0.5 µL of TaqMan 20× probe and 5.0 µL of 2× FastStart Essential DNA Probe Master Mix (Roche, Basel, Switzerland). Cycling conditions were 50 °C for 2 min, 95 °C for 10 min (initial denaturation), and 45 cycles of 95 °C for 15 s and at 62 °C for 1 min (annealing/extension).

### 2.7. Expression of lncRNAs and miR-200 Family in KIRC (ccRCC) from RNA Sequencing Datasets Using the Cancer Genome Atlas

To verify the expression of the analyzed transcripts, we utilized RNA sequencing data from the kidney renal clear cell carcinoma (KIRC) project from The Cancer Genome Atlas (TCGA) (accessed on 9 June 2023). After identifying and removing duplicate samples during preprocessing, 511 unique tumor samples remained, all corresponding to ccRCC. The cBioPortal interactive online database was used to access the data. Read per million (RPM) mapped miRNA isoforms for miRNA experiments and fragments per kilobase of transcript per million mapped reads in the upper quartile (FPKM-UQ) were obtained as normalized gene counts (level 3 data). Data were presented in box plots as RPM and FPKM-UQ for miRNA and lncRNA experiments, respectively. The R programming package was used for statistical analyses of correlations. Unfortunately, no samples of sRCC were identified in the database.

### 2.8. Statistical Analysis

Results were expressed as relative gene expression using the ΔCq method. At first, all Cqs were corrected for PCR efficiencies for miRNAs and RGs. Second, the average value of Cq was calculated from technical replicates for each biological replicate. Next, the geometric mean of average Cq values for the RGs was subtracted from the average Cq values of the genes of interest (GOIs, e.g., miRNA, lncRNA) to obtain ∆Cq for each biological sample. The results were presented as box plots, where a lower value means higher expression of miRNA or lncRNA. For correlations in our own samples, Spearman rank-order correlation was used; for correlations in TCGA samples, the Pearson correlation coefficient was used. We additionally performed the Mann–Whitney test to analyze differences in the expression of the *miR-200* family and lncRNAs between TP, TC, VTT, sRCC-Ca, sRCC-Sa, ccRCC, and non-neoplastic renal cortical tissue. Statistical analysis of data was performed using SPSS version 27 (SPSS Inc., Chicago, IL, USA). Differences were considered significant at *p* values < 0.05.

## 3. Results

### 3.1. Patients and Tissue Samples

**Figure 1:**
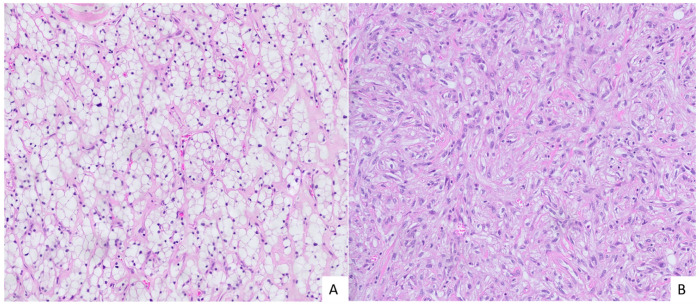
Morphology of sarcomatoid renal cell carcinoma (sRCC). (**A**) Carcinomatous component of sRCC: nests of cells with abundant clear cytoplasm with small nuclei. A network of small vessels surrounds tumor nests, a characteristic feature of clear cell RCC (HE, orig. magnification 20×). (**B**) Sarcomatous component of sRCC: malignant spindle cells with pleomorphic nuclei growing in short irregular fascicles (HE, orig. magnification 20×).

**Figure 2:**
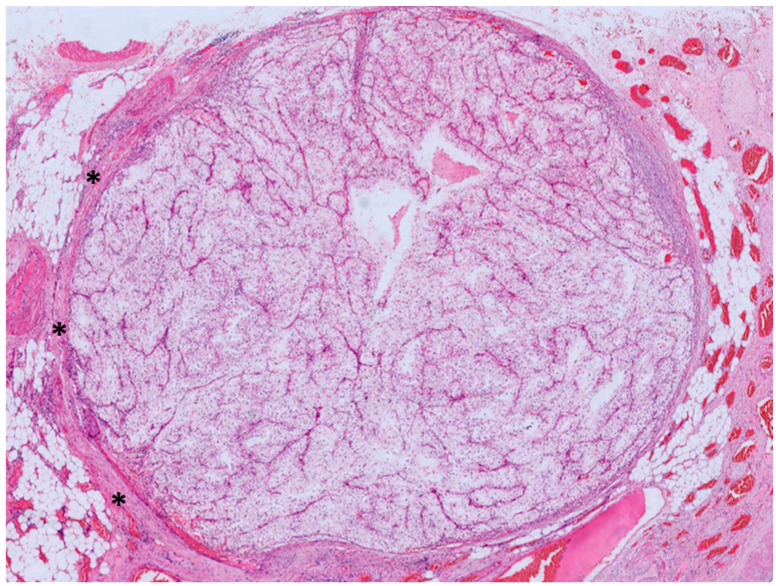
Vascular invasion in clear cell renal cell carcinoma (ccRCC): a venous tumor thrombus filling and distending the lumen of a segmental renal vein in the renal hilum. The vessel wall is highlighted by asterisks (HE, orig. magnification 2×).

In total, we analyzed 42 samples from 25 patients. There were 20 males and 5 females, with a mean age of 62.9 ± 9.6. In early ccRCC, partial nephrectomies were performed in all but one instance. For advanced ccRCC, all patients underwent total nephrectomies, while in the sRCC group, all but one underwent total nephrectomies. In the early ccRCC group, all tumors were classified as pT1a. In the advanced ccRCC group, nine cases were pT3a and one was pT3b. In the sRCC group, tumor stages included pT1a (*n* = 1), pT2a (*n* = 1), pT3a (*n* = 5), and pT4 (*n* = 1). Detailed information is provided in Table 1. The sampling distribution among patients is presented in Table S1. Morphological features are presented in Figure 1 and Figure 2.

### 3.2. Indentification of Target lncRNAs

#### 3.2.1. Identified lncRNAs with Potentially Regulatory Function Toward *miR-200* Family

Using PubMed, we searched for lncRNAs that could potentially regulate the *miR-200* family. We identified 127 lncRNAs that could regulate any of the *miR-200* family members. The list of all identified lncRNAs with target miRNA of the *miR-200* family is summarized in Table S2. Among these 127 lncRNAs, we searched for predesigned TaqMan probes with a Small RNA assay to perform validation on RCC samples. We identified 31 such lncRNAs and performed expression analysis in our cohort. A detailed list of these 31 lncRNAs is summarized in Table S3 and in Table 2.

#### 3.2.2. miRNA-lncRNA Interactions Identified from Cross-Database Search

Our cross-database search returned differing numbers of lncRNA targets for each *miR-200* family member. The numbers of lncRNA targets are presented in Table 3. Detailed results with specific lncRNAs are presented in Tables S4–S6.

Intersecting all three databases per miRNA yields the results shown in Table 4. The three-way intersection contained two lncRNAs for *miR-200a* and three for each of the remaining *miR-200* members.

We also performed within-database *miR-200* family intersections to identify lncRNAs reported for all five *miR-200* members within a single database. This yielded 15 lncRNAs in DIANA-LncBase v3, 6 in ENCORI, and 22 in RNA Interactome (Table 5).

Interestingly, *MALAT1* was the only lncRNA common both to all *miR-200* family members and to all three databases (DIANA-LncBase v3, ENCORI, and RNA Interactome).

### 3.3. Expression of miR-200 Family, Their Regulatory lncRNAs, and Correlation Between Them in ccRCC and sRCC

#### 3.3.1. Expression of *miR-200* Family in Our Cohort of Patients

**Figure 3:**
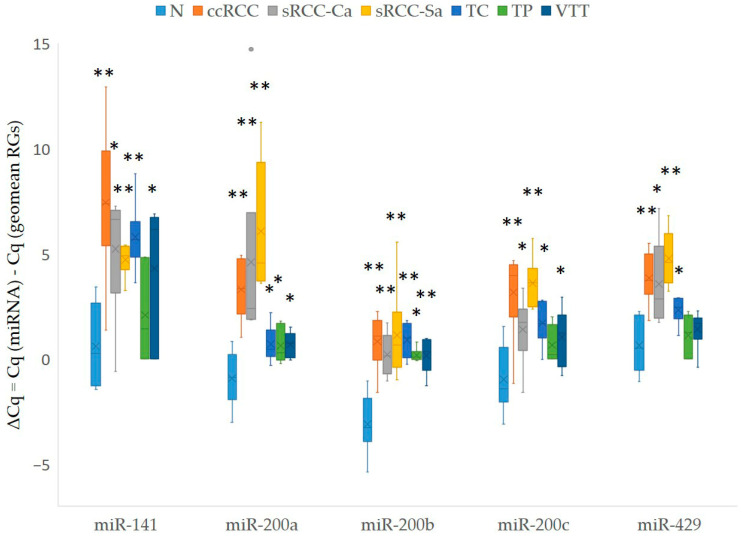
Expression of *miR-200* family in different entities of RCC and in different parts of RCC with renal vein invasion. Legend: ccRCC, clear cell renal cell carcinoma (*n* = 6); geomean, geometric mean; GOI, gene of interest; N, morphologically normal tissue adjunct to tumor (*n* = 6); n, number of biological replicates; RGs, reference genes; sRCC, sarcomatoid renal cell carcinoma; sRCC-Ca, carcinomatous component of sRCC (*n* = 6); sRCC-Sa, sarcomatoid component of sRCC (*n* = 6); TC, tumor center (*n* = 6); TP, tumor periphery (*n* = 6); VTT, venous tumor thrombus (*n* = 6); *, *p* < 0.05; **, *p* < 0.01. Each biological replicate was performed in two technical replicates, and, for ΔCq calculation, for each biological replicate, the average value of two technical replicates was used.

We observed consistent downregulation of all members of the *miR-200* family compared to the non-neoplastic renal cortex, and this downregulation was observed in all tumor samples, including early ccRCC, TP, VTT, and TC of advanced ccRCC and the carcinomatous and sarcomatoid component of sRCC. The results are summarized in Figure 3. The statistical analysis of the expression of the *miR-200* family between different groups of samples was limited by the small sample size within each tested group (*n* = 6); therefore, the results are presented in Table S7.

#### 3.3.2. Expression of lncRNAs in Our Cohort of Patients

**Figure 4:**
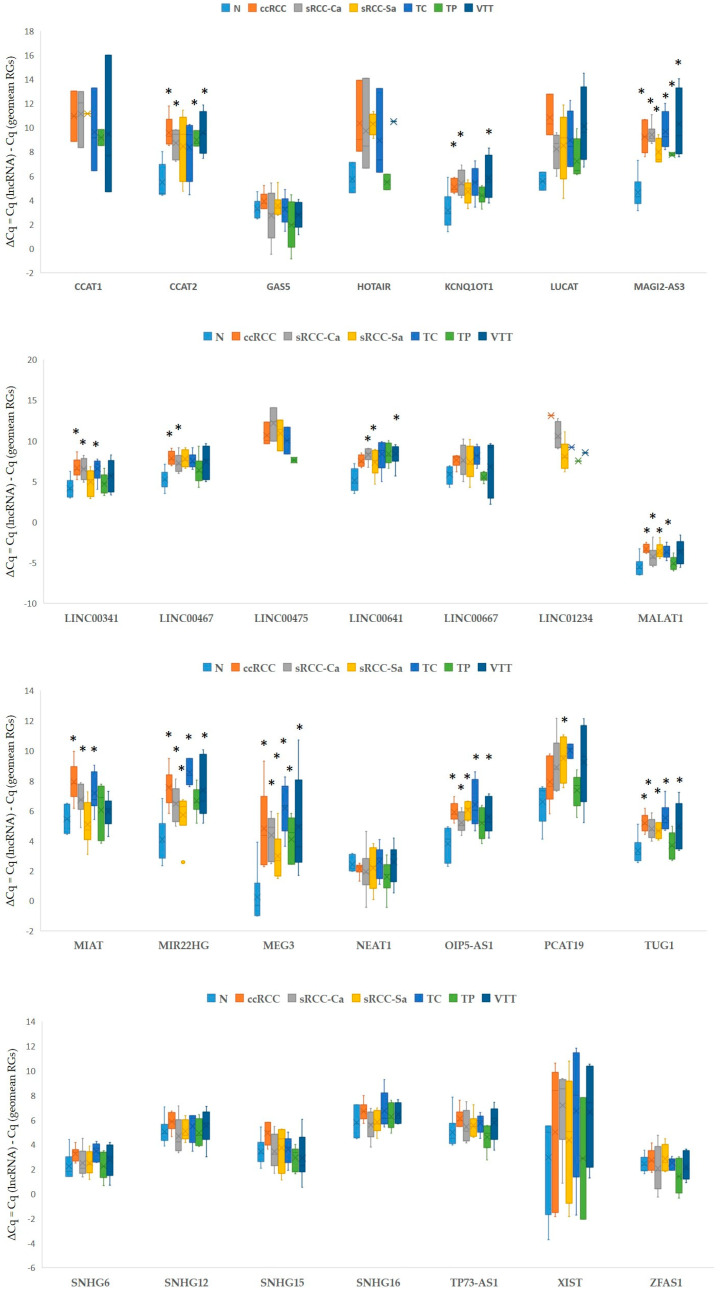
Expression of analyzed lncRNAs as potential regulators of *miR-200* family in early ccRCC, advanced ccRCC, and sRCC. Legend: ccRCC, clear cell renal cell carcinoma (*n* = 6); geomean, geometric mean; GOI, gene of interest; N, morphologically normal tissue adjunct to tumor (*n* = 6); n, number of biological replicates; RGs, reference genes; sRCC, sarcomatoid renal cell carcinoma; sRCC-Ca, carcinomatous component of sRCC (*n* = 6); sRCC-Sa, sarcomatoid component of sRCC (*n* = 6); TC, tumor center (*n* = 6); TP, tumor periphery (*n* = 6); VTT, venous tumor thrombus (*n* = 6); *, *p* < 0.05. Each biological replicate was performed in 2 technical replicates, and, for ΔCq calculation, for each biological replicate, the average value of two technical replicates was used.

Out of 31 tested lncRNAs, 13 were expressed in all tested samples. Six lncRNAs were expressed in most samples (38 to 41), five in over half of the samples (28 to 35), and four lncRNAs in a smaller number of samples (13 to 19). One lncRNA previously linked to *miR-200* regulation was below the detection limit (*MAGI1-IT1*); two miRNAs were omitted from further analysis due to article retractions (*TUNCAR* and *UCA1*). The results are summarized in Figure 4. The statistical analysis of the expression of 31 lncRNAs between different groups of samples is shown solely in Table S7, due to the small sample size in each tested group (*n* = 6).

#### 3.3.3. Correlation Between Expression of *miR-200* Family and Expression of Potentially Regulatory lncRNAs

Of the 31 analyzed lncRNAs, the expression of 17 lncRNAs showed a statistically significant correlation with the expression of at least one member of the *miR-200* family. Notably, the expression of two miRNAs, *miR-200b* and *miR-141*, correlated with the expression of more than half of these lncRNAs: *miR-200b* correlated with the expression of 16 lncRNAs, and *miR-141* correlated with the expression of 11 lncRNAs. In contrast, the expression of the other three miRNAs, *miR-200a*, *miR-200c*, and *miR-429*, each correlated with fewer lncRNAs: four, four, and seven lncRNAs, respectively.

**Figure 5:**
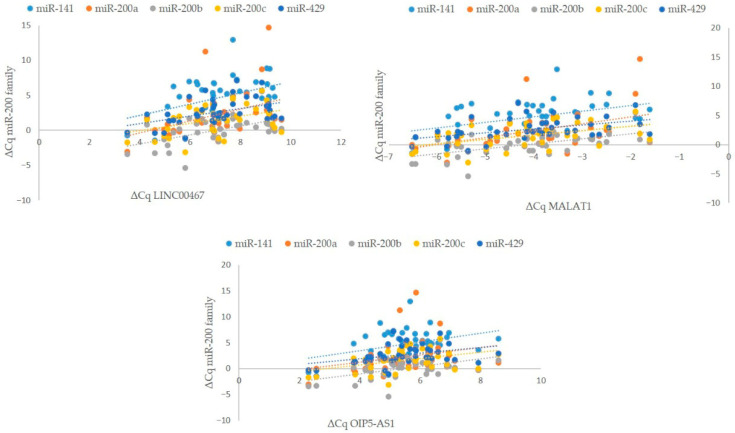
Correlation between lncRNA (*LINC00467*, *MALAT1*, and *OIP5-AS1*) and *miR-200* family expression in RCC (*n* = 42). Legend: ∆Cq, delta quantitation cycle (geometric mean of Cq of reference genes subtracted from Cq of gene of interest, e.g., lncRNA/miRNA); n, number of biological replicates. Each biological replicate was performed in 2 technical replicates, and, for ΔCq calculation, for each biological replicate, the average value of two technical replicates was used.

Conversely, the expression of six lncRNAs correlated with the expression of only a single member of the *miR-200* family—*miR-200b* in five cases and *miR-200a* in one. The expression of four lncRNAs correlated with the expression of two members of the *miR-200* family, the expression of three lncRNAs correlated with the expression of three members of the *miR-200* family, and the expression of one lncRNA correlated with the expression of four members of the *miR-200* family. Finally, the expression of three lncRNAs, namely, *LINC00467, MALAT1*, and *OIP5-AS1*, correlated with the expression of all five members of the *miR-200* family. The results are summarized in Table S8 and Figure 5.

### 3.4. Expression of miR-200 Family, Their Potentially Regulatory lncRNAs, and Correlation Between Them in KIRC from TCGA

#### 3.4.1. Expression of *miR-200* Family in KIRC Samples from TCGA

**Figure 6:**
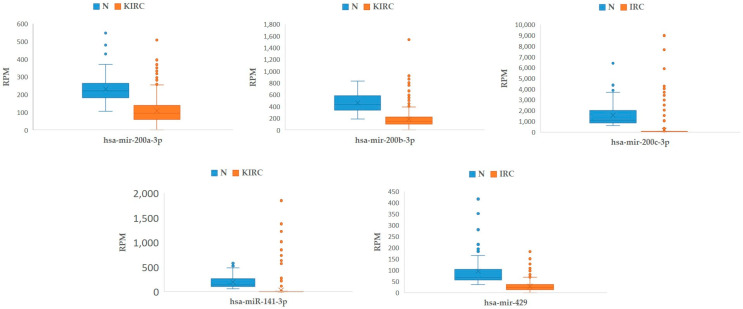
Expression of *miR-200* family in KIRC samples from TCGA. Legend: KIRC, kidney renal clear cell carcinoma (*n* = 511); N, morphologically normal tissue adjunct to tumor (*n* = 71); n, number of biological replicates; RPM, reads per million; TCGA, The Cancer Genome Atlas.

When analyzing the number of normalized transcripts of the *miR-200* family in carcinomatous tissue compared to non-carcinomatous tissue of KIRC, we observed a consistently smaller number of transcripts in carcinomatous tissue compared to non-carcinomatous tissue. The results are in accordance with those observed in our samples and are summarized in Figure 6.

#### 3.4.2. Expression of lncRNAs in KIRC Samples from TCGA

When analyzing the expression of all 127 identified lncRNAs with regulatory function toward any of the *miR-200* family members using KIRC and TCGA, we observed that 86 lncRNAs were expressed in carcinomatous and non-carcinomatous tissue. Of 31 lncRNAs quantified using qPCRs on our samples, transcripts were detected/annotated for 24 lncRNAs in carcinomatous and non-carcinomatous KIRC samples. Combining the results of KIRC samples from TCGA and our samples, we confirmed the expression of 92 identified lncRNAs in renal/kidney tissue.

We further analyzed whether the expression of *LINC00467, MALAT1*, and *OIP5-AS1* was similar to that observed in our cohort of samples, and we observed a similar trend of expression.

**Figure 7:**
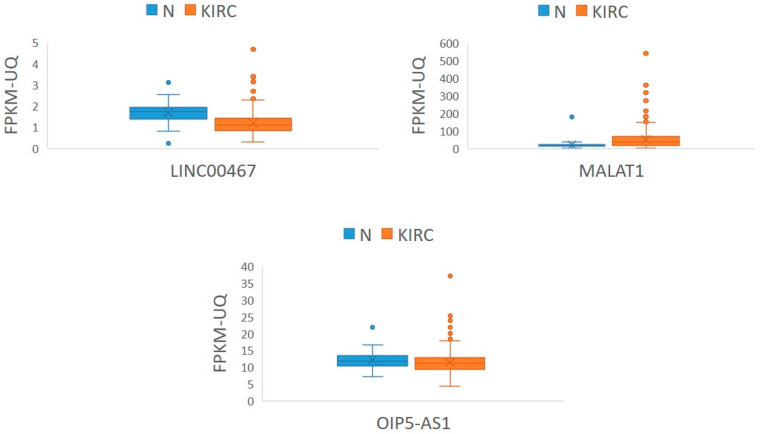
Expression of *LINC00467, MALAT1*, and *OIP5-AS1* in KIRC samples from TCGA. Legend: FPKM-UQ, fragments per kilobase of transcript per million mapped reads in the upper quartile; KIRC, kidney renal clear cell carcinoma (*n* = 511); N, morphologically normal tissue adjunct to tumor (*n* = 71); n, number of biological replicates; RPM, reads per million; TCGA, The Cancer Genome Atlas.

The results are summarized in Tables S9 and S10 and Figure 7.

#### 3.4.3. Correlation Between *miR-200* Family and Potentially Regulatory lncRNAs

We have calculated the correlations between the *miR-200* family and 86 expressed lncRNAs in carcinoma and non-carcinoma tissue in KIRC. The expression of five lncRNAs, namely, *CRNDE*, *LINC00365*, *MSC-AS1*, *SFTA1P*, and *TRAF3IP2-AS1*, were in correlation with the expression of all members of the *miR-200* family.

To validate the findings obtained from our cohort, we performed an additional correlation analysis using tumor and morphologically normal samples from KIRC, applying statistical evaluation with SPSS. This analysis confirmed a weak positive correlation between the expression of the *miR-200* family and *LINC00467*, a very weak positive correlation between expression of *OIP5-AS1* and expression of *miR-200a/b/c,* and a very weak negative correlation between expression of *MALAT1* and expression of *miR-200c*.

The results are summarized in Table S11.

## 4. Discussion

miRNAs are encoded as inter- or intragenic units. Intergenic miRNAs are encoded as single genes or as clusters, leading to co-regulation and co-transcription of certain clusters [89], and their transcription in the form of independent units, as a monocistronic, bicistronic or polycistronic primary transcript [90]. Although the functional roles of miRNAs vary, their primary mechanism of action in mammals is believed to involve the inhibition of mRNA translation by base-pairing with the 3′ UTR of target mRNAs. Each miRNA is predicted to have as many as 200 target genes, and it is also common for mRNA to be regulated by multiple miRNAs. This cooperative action, either through multiplicity (multiple identical miRNAs) or through cooperativity (different miRNAs), enhances the efficiency of translational inhibition and increases the specificity of miRNAs. Moreover, combinatorial control of gene expression may arise from a set of coordinately expressed miRNAs [91]. The *miR-200* family comprises five members: *miR-200a*, *miR-200b*, *miR-200c*, *miR-141*, and *miR-429*. This family can be classified into two categories based on chromosomal location: Cluster I includes *miR-200a*, *miR-200b*, and *miR-429*, while Cluster II consists of *miR-141* and *miR-200c*. Additionally, a classification based on seed sequences that bind to the 3′ UTR of target genes further divides the family: Cluster I includes *miR-200b*, *miR-200c*, and *miR-429*, whereas Cluster II comprises miR-*200a* and *miR-141*. It is hypothesized that members of the same miRNA family are distributed across different genomic locations, which may enhance the spatial, temporal, and tissue-specific regulation of target gene expression. This regulation is further influenced by histone modifications [6,7]. Beyond miRNAs, other ncRNAs play crucial roles in the pathogenesis of cancer and various diseases and can also regulate miRNA expression [7]. In our previous research, we summarized the lncRNAs that function as competitive endogenous RNAs, thereby modulating *miR-200* expression and contributing to the metastatic phenotype in different cancers [9]. In the present study, we identified three lncRNAs, *MALAT1, OIP5-AS1*, and *LINC00467*, as potential regulators of *miR-200* family expression in ccRCC.

Several studies have demonstrated the upregulation of long intergenic non-coding RNA 467 (*LINC00467*) across various cancer types, suggesting its potential oncogenic role (reviewed in [92]). Current data indicates that its primary mode of action involves functioning as a ceRNA, effectively sponging multiple miRNAs. In urological malignancies, *LINC00467* has been found to be upregulated in bladder carcinoma, where it regulates tumor cell proliferation and invasiveness through the NF-κB signaling pathway. Gao et al. [43] showed that *LINC00467* negatively regulates the expression of *miR-200a* in glioma cell lines, while promoting *E2F3* expression. To the best of our knowledge, our study is the first to suggest the role of the entire *miR-200* family as an indirect or direct target of *LINC00467* in RCC.

Analysis of various carcinomas shows that OIP5 antisense transcript 1 (*OIP5-AS1*) is implicated in the oncogenesis of these carcinomas and that OIP5 is highly expressed in neural tissues [93]. Its role appears to be contradictory, with some studies reporting its tumor-suppressive function (reviewed in [94]). Additionally, Liu et al. [95] observed a downregulation of *OIP5-AS1* in endometrial carcinoma and identified *miR-200c* as its target. In the context of urological malignancies, *OIP5-AS1* is upregulated and functions as an oncogene, correlating with poorer survival outcomes [96,97]. While Zhang et al. [98] demonstrated an oncogenic role of *OIP5-AS1* in bladder carcinoma and identified *miR-217* as a direct target, Feraydoon et al. [99] reported a tumor-suppressive role of this transcript. Interestingly, a study by Fu et al. [100] showed that by binding to *miR-30c-5p* in diabetic nephropathy, *OIP5-AS1* induces EMT and renal fibrosis, highlighting the diverse effects of lncRNAs under physiological and pathological conditions.

In various cancer types, there is increasing evidence to suggest that *MALAT1* is abnormally expressed, suggesting that *MALAT1* functions as an oncogene through multiple biological mechanisms, including its role as a ceRNA that binds to various miRNAs [101,102]. In non-small cell lung carcinoma, *MALAT1* facilitates disease progression by modulating the *miR-200a-3p*/PDL1 axis [103]. The interplay between *MALAT1* and members of the *miR-200* family has also been demonstrated in endometrioid endometrial carcinoma [104], anaplastic thyroid carcinoma [105], and hepatocellular carcinoma [106]. In RCC tissues, *MALAT1* is highly expressed, and acts as a sponge for the *miR-200* family [54], leading to the induction of zinc finger E-box-binding homeobox 2 (*ZEB2*). Furthermore, Huang et al. [107] provided evidence that *MALAT1* can sponge *miR-205*, facilitating proliferation and invasion in RCC. These results emphasize the diverse pathways through which *MALAT1* can contribute to the progression of RCC.

Our analysis revealed a positive correlation between the expression of the *miR-200* family and investigated lncRNAs. There are several potential explanations that may account for this observation. Expression profiling of many ncRNAs in various normal and diseased tissues has revealed unique spatial and temporal expression patterns [108]. Many ncRNAs exhibit tissue-specific and/or cell type-specific expression [109]. Given the diversity of genes and expression patterns, it is reasonable to propose that each cell type might have a distinct miRNA expression profile at different developmental stages. Therefore, it is not surprising that our analysis revealed an inverse correlation, in contrast with the patterns observed between lncRNAs and the *miR-200* family in in vitro and in vivo ‘’non-RCC’’ studies.

Another explanation for the observed positive correlation could lie in the nature of lncRNAs. Large intergenic ncRNAs (lincRNAs) tend to be modestly conserved and can both up- and downregulate the expression of hundreds of genes, including miRNAs. Additionally, about 25% of enhancers co-localize with RNAPII sites, suggesting that some enhancer RNAs (eRNAs) are also transcribed [110] and play key roles in epigenetic gene regulation in mammalian cells. Generally, lncRNAs have been implicated in gene-regulatory roles, influencing nearly every step in the life cycle of genes, including transcription, mRNA splicing, and RNA decay and translation [108]. Therefore, a function observed in one cell type (e.g., non-RCC) may not necessarily be conserved or established in another type (e.g., RCC).

Finally, a key distinction between epithelial-derived carcinomas and ccRCC is that ccRCC originates from the malignant transformation of proximal tubular cells [111], which embryologically originate from mesenchymal tissue, specifically the metanephric mesenchyme [112]. Notably, both ccRCC and normal proximal tubular cells can co-express epithelial and mesenchymal cadherins [19,113,114]. This may partially explain the observed differences in the expression trends of the investigated EMT markers and their regulators compared to carcinomas derived from epithelial tissue.

The observed discrepancies observed between our samples and those from The Cancer Genome Atlas (TCGA) can be attributed to at least two factors. First, there is a difference in the utilized samples. The TCGA dataset includes samples from various projects, employing different isolation methods from either FFPE or fresh-frozen tissue, and encompasses ccRCC samples of different stages and grades. In contrast, our samples include not only ccRCC but also sarcomatoid RCC (sRCC), along with vascular tumor thrombus (VTT) samples from ccRCC. Second, we employed qPCR, while TCGA data are based on next-generation sequencing (NGS). Both approaches have inherent differences related to the targets used. Many commercial NGS platforms have emerged, achieving base accuracy rates as high as 99.99% due to the vast amount of information they process. However, this can create a bottleneck in analysis, as bioinformatics expertise is required to interpret the data accurately. Additionally, potential biases can occur, such as PCR bias during library preparation or sequencing; high GC content or secondary structure fragments are less likely to be amplified, and it can be challenging to determine whether the number of repetitions reflects the actual sample or is a deviation caused by PCR. In contrast, qPCR involves reverse transcription of RNA into complementary DNA (cDNA), followed by amplification using real-time fluorescence. There is a linear relationship between the cycle quantitation (Cq value) of each template and the logarithm of the starting copy number of the template. This quantification process is highly automated, and the quality standards are relatively stable, ensuring the accuracy of the results. Consequently, qPCR is considered an established method that is both sensitive and specific [115]. In parallel, data from multiple databases—including DIANA, ENCORI, and RNA Interactome—support the involvement of certain lncRNAs (e.g., *MALAT1*, *OIP5-AS1*) as putative regulators or targets of the *miR-200* family in RCC.

However, our study has several limitations. First, we did not perform functional validation (e.g., gain- and loss-of-function assays, in vitro and in vivo experiments) for lncRNAs whose expression correlated with at least one member of the *miR-200* family. While some of these lncRNAs have already been previously validated in other cancer types, they have not yet been validated in RCC cell lines. Second, the relatively smaller sample size, resulting from the analysis of a larger number of lncRNA transcripts (*n* = 31) may have limited the statistical power of our findings. However, this limitation could be partially mitigated by incorporating KIRC data from the TCGA cohort. Finally, further research is needed to explore the clinical utility of the *miR-200* family, including multi-center validation studies.

## 5. Conclusions

ncRNAs primarily serve regulatory functions, do not encode proteins, and are represented by a diverse class of RNA molecules. The *miR-200* family is a well-known inhibitor of EMT. Our findings suggest that, in RCC, the expression of the *miR-200* family might be at least partially in correlation with the expression of certain lncRNAs. Thus, investigating co-expression patterns between different classes of ncRNAs in RCC (e.g., between *miR-200* family and lncRNAs) may serve as a starting point and opportunity for the development of experimental and cell-line based research settings to confirm interaction between certain lncRNAs and the *miR-200* family in RCC as well. Such investigations might provide deeper insights to aid our understanding of the progression of RCC to the more aggressive form of the disease that occurs in up to 30% of patients (e.g., the metastatic form and sRCC).

## Figures and Tables

**Table 1:** Patient and tumor characteristics with tissue sample regions by group.

Group	Age (Mean ± SD)	Male/Female	Tumor Size (Mean)	Nuclear Grade	Tissue Samples
Early ccRCC	62.7 ± 4.6	4:3	2.2 ± 0.7	2	Non-Tumorous Kidney (*n* = 3)
					Carcinoma (*n* = 6)
Advanced ^†^ ccRCC	64.6 ± 8.9	10:0	6.3 ± 2.2	2 (*n* = 2)	Non-Tumorous Kidney (*n* = 3)
					Carcinoma–Central Part (*n* = 6)
				3 (*n* = 7)	Carcinoma–At Hilum (*n* = 6)
					Venous Tumor Thrombus (*n* = 6)
sRCC ^§^	61.1 ± 13.4	6:2	8.9 ± 3.2	4	Carcinomatous Component (*n* = 6)
					Sarcomatous Component (*n* = 6)

Legend: ccRCC, clear cell renal cell carcinoma; early, pT1; ^†^ advanced ccRCC, pT3 ccRCC with venous invasion; ^§^ sRCC, sarcomatoid RCC. TNM staging was performed according to the latest TNM classification of renal tumors [26].

**Table 2:** Currently known interactions of 31 analyzed lncRNAs with *miR-200* family members in various cancer types and disease states.

lncRNA	Target miRNA	Associated Disease	References
*CCAT1*	*miR-200b*	Anaplastic Thyroid Carcinoma	[33]
*CCAT2*	*miR-200b*	Esophageal Squamous Cell Carcinoma	[34]
*GAS5*	*miR-200* family	COVID-19	[35]
*HOTAIR*	*miR-141, miR-200a*, *miR-200b*, *miR-200c*	RCC Cell Lines, Gastric Carcinoma Cell Line, Ovarian Carcinoma	[36,37,38]
*LINC00341*	*miR-141*	Osteoarthritis	[39]
*KCNQ1OT1*	*miR-141/-200a/b*	Osteoporosis, Cerebral Ischemic Stroke, Skin Wound Healing	[40,41,42]
*LINC00467*	*miR-200a*	Glioma Cell Line	[43]
*LINC00475*	*miR-141*	Glioma	[44]
*LINC00641*	*miR-429*	Gastric Cancer	[45]
*LINC00667*	*miR-200b/c/429*	Wilms Tumor, Esophageal Carcinoma, Breast Carcinoma, Cholangiocarcinoma	[46,47,48,49]
*LINC01234*	*miR-429*	Breast Carcinoma	[50]
*LUCAT1*	*miR-200c*	Osteosarcoma	[51]
*MAGI2-AS3*	*miR-141/200a*	Gastric Carcinoma	[52]
*MALAT1*	*miR-200* family	RCC, Lung Carcinoma	[53,54,55]
*MEG3*	*miR-141*, *miR-200a/c*	Breast Carcinoma, Lung Carcinoma Cell Line	[56]
*MIAT*	*miR-141*	Gastric Carcinoma, Osteosarcoma	[57,58]
*MIR22HG*	*miR-141*	Endometrial Carcinoma, Anaplastic Thyroid Carcinoma	[59,60,61]
*NEAT1*	*miR-141*, *miR-200b*	Endometriosis, Breast Carcinoma, Melanoma	[62,63,64]
*OIP5-AS1*	*miR-429*, *miR-200c*	Lung Adenocarcinoma, Pancreatic Adenocarcinoma, Endometrial Carcinoma	[65,66]
*PCAT19*	*miR-429*	Gastric Carcinoma, Lung Adenocarcinoma	[67,68]
*SNHG6*	*miR-141*, *miR-429*	Osteosarcoma, Wilms Tumor	[69]
*SNHG12*	*miR-200c*, *miR-429*	Renal Cell Carcinoma, Lung Adenocarcinoma	[70,71]
*SNHG15*	*miR-141*, *miR-200a*	Osteosarcoma, Papillary Thyroid Carcinoma, Hepatocellular Carcinoma, Nasopharyngeal Carcinoma	[72,73,74]
*SNHG16*	*miR-200a*	Colorectal Carcinoma	[75]
*TP73-AS1*	*miR-141*, *miR-200a*	Hepatocellular Carcinoma, Pancreatic Ductal Adenocarcinoma, Breast Carcinoma	[76,77,78]
*TUG1*	*miR-138*, *miR-141*	Renal Interstitial Fibrosis Cervical Carcinoma, Hepatocellular Carcinoma, Pancreatic Carcinoma	[79,80,81,82]
*XIST*	*miR-141*, *miR-200a*, *miR-200b, miR-200c*, *miR-429*	Non-Small Cell Lung Carcinoma, Hepatocellular Carcinoma, Bladder Carcinoma, Hepatocellular Carcinoma, Cervical Carcinoma	[83,84,85,86,87]
*ZFAS1*	*miR-200b*, *miR-200c*	Colorectal Carcinoma	[88]

Note: For *MAGI1-IT1, TUNCAR*, and *UCA1*, the suggested target miRNAs were *miR-200a, miR-200a*, and *miR-200c* in ovarian cancer, glioma, and hemangioma, respectively; however, all articles were retracted.

**Table 3:** Number of lncRNA targets per *miR-200* family member in DIANA-LncBase v3, ENCORI, and RNA Interactome databases.

Database	*miR-200a*	*miR-200b*	*miR-200c*	*miR-141*	*miR-429*
DIANA-lncBase v3	178	140	204	229	174
ENCORI	26	23	24	26	24
RNA Interactome	116	123	118	120	131

**Table 4:** Common lncRNA targets across DIANA-lncBase v3, ENCORI, and RNA Interactome databases for each *miR-200* family miRNA.

*miR-200a*	*miR-200b*	*miR-200c*	*miR-141*	*miR-429*
*MALAT1*	*MALAT1*	*MALAT1*	*MALAT1*	*MALAT1*
*SNHG16*	*OIP5-AS1*	*OIP5-AS1*	*SNHG16*	*OIP5-AS1*
	*XIST*	*XIST*	*XIST*	*XIST*

**Table 5:** Common lncRNA targets across the *miR-200* family identified within each database—DIANA-LncBase v3, ENCORI, and RNA Interactome.

DIANA-lncBase v3	ENCORI	RNA Interactome
*AC010342.1*	*AL049840.5*	*AQP4-AS1*
*AL118506.1*	*KCNQ1OT1*	*ASTN2-AS1*
*AP000926.1*	*MALAT1*	*ATP2B1-AS1*
*GAS5*	*NEAT1*	*ATXN8OS*
*MALAT1*	*OIP5-AS1*	*C5orf56*
*NAP1L1P3*	*XIST*	*DRAIC*
*NEAT1*		*H19*
*OIP5-AS1*		*HAND2-AS1*
*PVT1*		*LINC00341*
*RPL23AP73*		*LINC00461*
*SNHG15*		*LINC00667*
*TDGP1*		*LINC01140*
*XIST*		*LINC01312*
*XLOC_006069*		*LINC02120*
*XLOC_008185*		*LOC256880*
		*LOC285074*
		*LOC401021*
		*MALAT1*
		*MSC-AS1*
		*PART1*
		*TTTY4B*
		*ZEB1-AS1*

## Data Availability

All data generated or analyzed during this study are included in the article; further inquiries can be directed to the corresponding author.

## Supplementary Materials

The following supporting information can be downloaded at https://www.mdpi.com/article/10.3390/cancers17193123/s1, Table S1: The scheme of sampling distribution among patients; Table S2: The list of all identified lncRNAs with target miRNA of the *miR-200* family using PubMed; Table S3: TaqMan probes used for expression analysis; Table S4: miRNA–lncRNA pairs retrieved from DIANA-LncBase v3 database for the *miR-200* family; Table S5: miRNA–lncRNA pairs retrieved from ENCORI database for the *miR-200* family; Table S6: miRNA–lncRNA pairs retrieved from RNA Interactome database for the *miR-200* family; Table S7: Summary of statistically significant *miR-200* family members and their potentially regulatory/target lncRNAs in RCC; Table S8: Spearman correlations of analyzed lncRNA and miR-200 family RNAs; Table S9: Expression of selected lncRNA in normal kidney tissue from TCGA database; Table S10: Expression of selected lncRNA in KIRC tumor samples from TCGA database; Table S11: Correlations between lncRNAs and *miR-200* family miRNAs in TCGA normal and tumor samples.

## Abbreviations

The following abbreviations are used in this manuscript:

ccRCC	clear cell RCC
EMT	epithelial–mesenchymal transition
FFPE	formalin-fixed paraffin-embedded
FPKM-UQ	fragments per kilobase of transcript per million mapped reads—upper quartile
GOI	gene of interest
HIF	hypoxia-inducible factor
ISUP	International Society of Urologic Pathologists
KIRC	kidney renal clear cell carcinoma
LINC00467	large intergenic non-coding RNA 00467
lncRNA	long non-coding RNA
MALAT1	metastasis-associated lung adenocarcinoma transcript 1
miRNA	microRNA
mRNA	messenger RNA
ncRNA	non-coding RNA
OIP5-AS1	OPA-interacting protein 5 antisense transcript 1
Qpcr	quantitative polymerase chain reaction
RCC	renal cell carcinoma
RNA	ribonucleic acid
RG	reference gene
RPM	reads per million
RT	reverse transcriptase/transcription
sRCC	sarcomatoid RCC
TC	tumor center
TCGA	The Cancer Genome Atlas
TNM	tumor node metastasis
TP	tumor periphery
WHO	World Health Organization
VTT	venous tumor thrombus
